# Reimagining criminal accountability: microbial and omics perspectives in the evolution of legal responsibility

**DOI:** 10.1093/jlb/lsaf022

**Published:** 2025-10-28

**Authors:** Pragya Mishra, Alan C Logan, Susan L Prescott

**Affiliations:** Department of Law, University of Allahabad (A Central University), Prayagraj, 211002, Uttar Pradesh, India; Center for Justice and Mental Well-Being, Nova Institute for Health, 1407 Fleet St., Baltimore, MD 21231, USA; Center for Justice and Mental Well-Being, Nova Institute for Health, 1407 Fleet St., Baltimore, MD 21231, USA; School of Medicine, University of Western Australia, Perth, WA, 6009 Australia; School of Medicine, University of Maryland, Baltimore, MD 21201, USA

**Keywords:** legalome, neurolaw, auto-brewery syndrome, microbiology, diminished capacity

## Abstract

Recent advances in microbiome science and omics technologies are reshaping our understanding of human behavior, suggesting that microbial communities significantly influence cognition, impulse control, and aggression. Emerging studies in neuromicrobiology, including fecal transplant studies, are pointing toward a causal role for gut microbes and their metabolites in human cognition and behavior. This essay introduces the legalome—a framework integrating microbial perspectives, including microbiome and omics sciences, into the courts and larger criminal justice system. We argue that the legalome is on a trajectory that will move the field of neurolaw forward, and challenge traditional doctrines of mens rea and culpability. Drawing on recent court decisions related to auto-brewery syndrome, and neuro-microbiological research, we examine how subtle biological processes influence behavior in ways overlooked by current legal standards. Recent findings raise questions about criminal intent, biological determinism, and equitable access to scientific defenses. At the same time, emergent research also suggests potential for microbiome-based rehabilitative interventions. Despite methodological challenges, we advocate for interdisciplinary collaboration to harmonize biological research with legal principles, creating a more nuanced framework for justice in the twenty-first century. The legalome provides concrete implementation protocols and assessment tools that demonstrate practical utility for courts, practitioners, and policymakers.

## I. INTRODUCTION

Recent advances in microbiome science and omics technologies are fundamentally reshaping our understanding of human behavior.^1^ The gut microbiome refers to the trillion-member community of bacteria, fungi, viruses, bacteria, and archaea residing in the gastrointestinal tract. Omics technologies allow researchers to identify objective markers (eg the metabolome, gut microbe–produced metabolites) and pair them with aspects of cognition and behavior, and neuropsychiatric risk.^2^ Emerging empirical evidence suggests that microbial communities influence cognition and behavior, shaping justice outcomes.^3^ High-throughput sequencing, metabolomics, and advanced bioinformatics have begun to unveil intricate biochemical pathways through which gut microbes interact with the central nervous system, thereby challenging traditional legal doctrines of mens rea^4^ and criminal responsibility.^5^

Recent investigations have begun explicitly connecting these emerging scientific domains to forensic psychology, arguing that microbiome science necessitates a recalibration of legal standards.^6^ For instance, consider recent cases in the USA and Europe wherein driving while intoxicated (DWI) charges were dismissed after scientific evidence indicated that the defendants suffered from auto-brewery syndrome—a condition in which gut microbes ferment carbohydrates into ethanol, resulting in intoxication without external alcohol consumption.^7^ These highly publicized cases may serve as a harbinger of how advancing microbiome science could force reassessments of intent and volition. While the DWI cases involving auto-brewery syndrome have focused on operating machinery with blood alcohol content (BAC) above locally set limits, it is worth noting that cognition can be disturbed at BAC levels far below driving statutes, and that many perpetrators and victims of violent crime have elevated BAC.^8^

Beyond the international headlines on DWI dismissals, the term ‘auto-brewery’ may be better viewed as a metaphor for the legal aspects of gut microbiota. Why? Because advances in metabolomics are demonstrating that ethanol is joined by countless microbiota-dependent metabolites that can alter cognition and behavior.^9^ Volumes of animal studies and preliminary human research indicates that microbial influences on behavior likely exist on a continuum, potentially affecting cognition and behavior—including aggression,^10^ violence, and antisocial acts.^11^ The biophysiological mechanisms linking gut microbes and human behavior, including impulse regulation, are being illuminated at a rapid pace.^12^ The implications of these developments extend far beyond individual cases. As microbiome sequencing becomes more accessible, questions arise regarding potential socioeconomic disparities in accessing these emerging biological defenses.

In response to these emerging trends, this essay introduces the concept of the legalome—a novel framework that integrates cutting-edge microbiome science into the criminal justice system, including forensic psychiatry and the courts.^13^ Of course, forensic applications of microbiology—including those that might place a perpetrator at a crime scene,^14^ aid in human identification, estimate postmortem intervals,^15^ and help establish cause of death^16^—have been ongoing for several years.^17^ However, the legalome focuses on the potential explanatory power of microbes in the context of cognition and behaviors that might otherwise lead to justice involvement. Moreover, the legalome includes consideration of the ways in which the human microbiome, as a biological transducer of multiple environmental inputs (ranging from food insecurity to workplace burnout), might influence the cognition and behaviors of professionals operating within the criminal justice system.^18^

Much like the transformative impact of neuroimaging on legal discourse,^19^ the integration of microbiome and omics technologies necessitates a re-evaluation of legal principles surrounding intent and responsibility.^20^ At both ends of the justice system, understanding how microbial ecosystems influence human behavior offers transformative potential for both prevention and rehabilitation.^21^ Targeted interventions—from precision dietary programs to microbiome-based therapies—could eventually complement or even supplant traditional punitive measures with evidence-based, rehabilitative strategies.

If emergent research continues to grow more robust, the integration of microbiome science into legal practice will present significant ethical and practical challenges.^22^ Issues concerning the admissibility of emerging scientific evidence, privacy protections, and the implications of biological determinism require scrutiny.^23^ As these technologies become more prevalent, ensuring equitable access to microbiome-based defenses and interventions will be crucial.^24^ This essay argues that legal scholarship must now engage meaningfully with microbiome science and omics technologies. As research continues to elucidate the profound yet complex influence of microbial ecosystems on cognition and behavior, legal institutions must navigate this intersection in a manner that upholds both justice and ethical integrity.^25^ This approach acknowledges that integrating novel scientific paradigms into jurisprudential practice requires careful consideration of both ethical and methodological dimensions.^26^

## II. THE MICROBIAL REVOLUTION IN NEUROSCIENCE AND LAW

The human microbiome—comprising trillions of microorganisms such as bacteria, fungi, and viruses residing primarily in the gut—performs a vast array of biochemical functions.^27^ In addition to aiding digestion, these microbes synthesize neurotransmitters, modulate inflammatory responses, and influence neurodevelopmental pathways that shape cognition,^28^ mood,^29^ and impulse control.^30^ The advent of metagenomics and multi-omics technologies has revolutionized the study of the microbiome,^31^ providing unprecedented insights into its potential role in human behavior.^32^ While still an emerging field, these discoveries are beginning new challenges to forensic psychiatry and criminal law^33^ in ways that would have been inconceivable even a decade ago.^34^

The field of neuromicrobiology, which examines the multifaceted interactions between microbial communities and the nervous system, has particularly advanced our understanding of how the microbiome influences neural processes that underlie behavior.^35^

### II.A. Mechanisms Linking the Microbiome to Behavior

Recent scientific research has delineated three principal pathways through which microbial ecosystems exert their influence over the brain:^36^

#### 1. The Neurochemical Pathway

Certain bacterial strains produce gamma-aminobutyric acid (GABA), serotonin, dopamine, and short-chain fatty acids—neuroactive compounds that directly modulate cognitive and emotional regulation.^37^ These metabolites traverse the blood–brain barrier, impacting neural circuits that govern impulse control, aggression, and decision-making.^38^

#### 2. The Immune Pathway

Gut microbiota play a key role in regulating systemic inflammation, which in turn affects brain function.^39^ Dysbiosis, or microbial imbalance, can provoke chronic inflammation that impairs prefrontal cortical regulation and increases susceptibility to impulsive or violent behavior.^40^ For example, infections with *Toxoplasma gondii* have been linked to behavioral changes, including potential increases in aggression,^41^ illustrating how microbial influences may intersect with legal considerations of intent.^42^

#### 3. The Vagal Pathway

The vagus nerve serves as a bidirectional conduit between the gut and the brain, transmitting microbial signals that influence emotional regulation, stress responses, and risk-taking behaviors.^43^ Studies involving germ-free animals have shown that the absence of a microbiome leads to heightened anxiety and aberrant social behavior—effects that are reversible upon reintroduction of normal microbial populations.^44^

These mechanisms underscore a crucial insight: the microbiome is not a peripheral factor but a central determinant of cognitive and affective states.^45^ Disruptions in the gut-brain axis—whether due to antibiotic overuse, poor diet, or environmental factors—can result in behavioral dysregulation with direct legal implications.^46^

### II.B. The Forensic Significance of Microbial Dysbiosis

Emerging forensic research indicates that certain microbial profiles may be associated with increased impulsivity and aggression,^47^ prompting provocative questions about culpability and criminal responsibility.^48^ Preliminary studies of incarcerated populations have revealed differences in gut microbiome markers (vs. non-incarcerated controls^49^), and individuals convicted of violent offenses maintain distinct microbial signatures.^50^ In following neuroimaging work that demonstrates preexisting (early life) neuroanatomical variability predicts subsequent substance use initiation,^51^ there is a need to determine if early life microbial signatures can predict subsequent justice involvement and risk of incarceration.^52^

Recent research highlights the role of biological markers in aggression and justice involvement, including correlations between gut microbiota, metabolic byproducts, and behavioral regulation.^53^ For instance, in adults with schizophrenia, a distinct gut microbial profile appears to distinguish between non-violent patients and persons prone to impulsive aggression.^54^ These data provide preliminary evidence that gut microbial alterations may be associated with reactive aggression and impulse control dysfunction observed in criminal acts. They point toward a mechanistic pathway for the oft-observed relationships between low-grade inflammation (measured via systemic inflammatory markers^55^ such as cytokines and C-reactive protein^56^) and human aggression.^57^ That is, disturbances to the gut microbiome appear to play a central role in the maintenance of chronic low-grade inflammation that otherwise lowers the threshold for aggressive, antisocial, and/or violent behavior.^58^

Moreover, early intervention trials—primarily in preclinical models—suggest that modifying the gut microbiota through probiotic or dietary adjustments can reduce impulsivity and aggression. For instance, prebiotic supplementation in rodents led to significant reductions in stress-related and impulsive behaviors.^59^ Emergent human research indicates that targeting the microbiome with probiotics can reduce aggression,^60^^,^^61^ aggressive thoughts,^62^ and impulsivity.^63^ Such findings hold profound implications for rehabilitative justice, indicating that microbiome-targeted therapies^64^ could eventually serve as adjunct interventions^65^ for reducing aggression among perpetrators of domestic violence and other criminal offenses.

These developments challenge traditional models of criminal responsibility.^66^ Given that courts have begun to reconsider mens rea^67^ in cases involving brain tumors,^68^ epilepsy, or neurodevelopmental disorders,^69^ it may be timely for the legal system^70^^,^^71^ to evaluate whether emerging microbiome science should be accorded similar evidentiary status. The subsequent section will further explore this issue through the lens of a particularly compelling case study: auto-brewery syndrome,^72^ wherein microbial fermentation results in endogenous alcohol production and consequent intoxication without volitional alcohol consumption.^73^

## III. BEYOND INTOXICATION: AUTO-BREWERY SYNDROME AND MICROBIOME-BASED LEGAL DEFENSES

Recent cases in the USA and Europe demonstrate the legal significance of auto-brewery syndrome (ABS), wherein defendants have successfully argued this condition as a defense against driving while intoxicated charges.^74^^,^^75^ Although ABS remains rare, its successful invocation raises important questions about the intersection of microbiology and culpability.^76^ Recent clinical interventions have demonstrated that microbiome-targeted treatments, particularly fecal microbiota transplantation, can effectively resolve ABS symptoms, providing evidence of the microbial etiology of this condition.^77^

However, ABS represents only one manifestation of a broader issue: the influence of microbiome-mediated metabolic processes on cognition, decision-making, and volitional control.^78^ While courts have long recognized intoxication as a mitigating factor, emerging insights into microbial interactions with neurotransmitter systems suggest that blood alcohol concentration may represent just one facet of how microbiome activity influences behavior.^79^^,^^80^ This raises a critical question: should the law extend beyond traditional intoxication defenses to consider more subtle microbial influences on cognition and impulse control?^81^

### III.A. Evidence for Causal Links

Emerging scientific evidence points toward causality between gut microbes and behavior. Preclinical fecal transplant studies demonstrate that cognitive and behavioral disturbances associated with dysbiosis transfer from ‘donor’ animals to healthy ‘recipient’ animals, resulting in behaviors indicative of aggression, heightened anxiety, altered mood, and cognitive impairment.^82^^–^^85^ These behavioral changes are accompanied by alterations in gene expression and neurotransmitter levels that reflect existing evidence in human neuropsychiatric disorders.

Mendelian randomization studies provide additional support for causal relationships, identifying bacterial taxa with apparent causal roles in conduct disorder, schizophrenia, and addiction disorders.^86^^–^^88^ Such findings suggest that microbiome-mediated mechanisms may influence behaviors relevant to criminal justice involvement.

### III.B. Socioeconomic Dimensions and Access to Justice

The successful invocation of ABS as a legal defense reveals concerning disparities in access to scientific defenses. Establishing an ABS defense requires extensive medical documentation, specialized testing, and expert testimony—resources often inaccessible to many defendants.^89^ This raises profound questions about equal access to justice: if microbial influences on behavior constitute valid defenses, should their availability be limited by financial means?^90^

Given that socioeconomic deprivation increases dysbiosis risk through environmental stressors, food insecurity, and toxin exposure,^91^^,^^92^ and considering the established links between socioeconomic marginalization and justice involvement,^93^^,^^94^ microbiome-based defenses risk exacerbating existing inequalities in the criminal justice system.

### III.C. Beyond Ethanol: The Broader Implications

While ABS demonstrates clear causal links between gut microbes and involuntary intoxication via ethanol production, research reveals that ethanol is only one of numerous gut microbe–produced chemicals linked to cognitive and behavioral alterations.^95^ Elevated levels of microbially produced metabolites such as *p*-cresol and specific short-chain fatty acids have been associated with behaviors including unprovoked physical aggression and threat behaviors.^96^^,^^97^ These metabolites influence brain neurotransmission and synaptic architecture, suggesting that microbiome dysbiosis may contribute to specific behavioral patterns constituting elements of criminal offenses.^98^

Recent findings indicate that breath testing can capture gut microbial metabolites related to impulsivity and executive function, expanding potential forensic applications beyond traditional alcohol detection.^99^ These developments suggest that microbiome-mediated influences on criminal behavior extend far beyond the narrow scope of auto-brewery syndrome, necessitating broader legal consideration of microbial factors in assessments of culpability and mens rea.

## IV. CURRENT LEGAL STANDARDS FOR SCIENTIFIC EVIDENCE IN CRIMINAL LAW

Before proposing novel frameworks for microbiome evidence, it is essential to examine existing legal standards governing the admissibility of scientific evidence in criminal proceedings. This foundation reveals both the precedents that support microbiome integration and the gaps that necessitate new approaches.

### IV.A. Established Evidentiary Standards

#### 1. The Daubert–Frye Framework

The admissibility of scientific evidence in US criminal courts is primarily governed by two landmark standards. The Frye standard, established in 1923, requires that scientific techniques be ‘generally accepted’ within the relevant scientific community.^100^ However, the Supreme Court’s 1993 decision in *Daubert v. Merrell Dow Pharmaceuticals* fundamentally reshaped this landscape, establishing a more flexible framework that emphasizes scientific validity over general acceptance.^101^

Under Daubert, courts must evaluate scientific evidence based on several factors: whether the theory or technique can be and has been tested; whether it has been subjected to peer review and publication; the known or potential rate of error; the existence of standards controlling the technique’s operation; and whether it has attracted widespread acceptance within the relevant scientific community.^102^ This framework has proven adaptable to emerging scientific disciplines, providing a foundation for evaluating microbiome evidence.

#### 2. Application to Biological Evidence

Courts have successfully integrated various forms of biological evidence under these standards. DNA fingerprinting, initially controversial, gained widespread acceptance through demonstrated reliability and standardized protocols.^103^ More recently, courts have grappled with neuroimaging evidence, with mixed results depending on the specific application and scientific rigor.^104^ Genetic evidence for predisposition to violence has generally been rejected when used to predict future behavior but accepted when demonstrating diminished capacity in specific cases.^105^

### IV.B. Forensic Microbiology: Current Applications

Existing forensic applications of microbiology provide important precedents for microbiome evidence. Clarke et al. identify three primary categories: individual identification, geographical sourcing, and postmortem interval estimation.^106^ Courts have shown greater receptivity to applications with clear protocols and established error rates, particularly in cases involving

- **Environmental source tracking**: Linking suspects to crime scenes through microbial signatures on objects or in locations^107^
- **Postmortem interval estimation**: Using bacterial succession patterns to determine time since death^108^
- **Individual identification**: Matching microbial profiles from personal items to specific individuals^109^

However, these applications focus primarily on identification rather than behavioral causation, representing a significant gap that microbiome–behavior research aims to address.

### IV.C. Gaps in Current Standards

#### 1. Behavioral Causation versus Identification

Current forensic microbiology applications largely avoid questions of behavioral causation that are central to criminal responsibility. While courts readily accept microbial evidence for identification purposes, they lack established frameworks for evaluating claims that microbial factors influenced criminal behavior.^110^ This gap becomes particularly problematic when defendants argue that microbiome dysbiosis affected their capacity for rational decision-making.

#### 2. Temporal Considerations

Existing standards poorly address the temporal dynamics of microbiome evidence. Unlike DNA, which remains relatively stable, microbiome composition can fluctuate rapidly based on diet, stress, medication, and environmental factors.^111^ Courts lack guidelines for determining the temporal relevance of microbiome data to alleged criminal acts.

#### 3. Causation versus Correlation

Perhaps most significantly, current standards inadequately distinguish between correlational and causal relationships in biological evidence. While emerging research suggests causal links between microbiome composition and behavior, courts lack established frameworks for evaluating such claims in criminal contexts.^112^

##### 1. International Perspectives

Comparative analysis reveals varying approaches to biological evidence across jurisdictions. European courts have shown greater willingness to consider novel biological factors in criminal cases, as evidenced by successful auto-brewery syndrome defenses in Belgium.^113^ However, most jurisdictions lack specific protocols for microbiome evidence, creating inconsistencies in evaluation and acceptance.

The European Court of Human Rights has emphasized the importance of scientific reliability in criminal proceedings, establishing principles that could support microbiome evidence integration while maintaining rigorous standards.^114^ These international precedents suggest pathways for developing comprehensive microbiome evidence frameworks.

## V. THE LEGALOME FRAMEWORK: A SYSTEMATIC APPROACH TO MICROBIOME EVIDENCE

Building upon the gaps identified in current legal standards, this section presents the legalome framework—a systematic approach to integrating microbiome science into criminal justice proceedings. Rather than proposing wholesale changes to existing legal principles, the legalome provides structured protocols for evaluating microbiome evidence while maintaining essential safeguards against biological determinism.

### V..A. Core Principles of the Legalome Framework

#### 1. Graduated Admissibility Standards

The legalome framework establishes three tiers of microbiome evidence, each with distinct admissibility requirements:

**Tier 1: Identification Evidence**—Microbiome data used for individual identification or scene linking, subject to standard forensic protocols with established error rates and quality controls.^115^

**Tier 2: Contextual Evidence**—Microbiome data providing context for criminal behavior (eg demonstrating recent medication effects, environmental exposures, post-prandial chemical production) without claiming direct behavioral causation.^116^

**Tier 3: Causal Evidence**—Microbiome data claiming direct causal influence on criminal behavior, subject to heightened scrutiny including demonstration of temporal proximity, established biological pathways, and exclusion of alternative explanations.^117^

#### 2. Scientific Reliability Requirements

Each tier incorporates specific reliability standards adapted from Daubert principles:

- **Standardized collection protocols** ensuring sample integrity and chain of custody
- **Validated analytical methods** with known error rates and quality controls
- **Peer-reviewed supporting research** demonstrating relevant biological pathways
- **Independent replication** of key findings by multiple research groups
- **Statistical significance testing** with appropriate controls for confounding variables.^118^

### V.B. Implementation Protocols

#### 1. Pre-Trial Evaluation

The framework requires specialized pre-trial hearings for Tier 2 and 3 microbiome evidence, conducted by judges with relevant scientific training or assisted by court-appointed experts.^119^ These hearings evaluate

1. **Scientific validity** of the proposed evidence under Daubert standards
2. **Temporal relevance** between microbiome sampling and alleged criminal acts
3. **Causal plausibility** based on established biological mechanisms
4. **Alternative explanations** and their relative likelihood
5. **Probative value** versus potential for prejudice or confusion^120^

#### 2. Expert Testimony Standards

The framework establishes specific qualifications for expert witnesses presenting microbiome evidence:

- **Advanced training** in relevant microbiome sciences (microbiology, bioinformatics, or related fields)
- **Research experience** in microbiome–behavior relationships
- **Forensic training** in legal standards and courtroom procedures
- **Continuing education** requirements to maintain current knowledge^121^

#### 3. Jury Instructions

Recognizing the complexity of microbiome science, the framework includes standardized jury instructions addressing

- **Limitations of microbiome evidence** and distinction between correlation and causation
- **Individual variability** in microbiome composition and response
- **Temporal considerations** affecting evidence interpretation
- **Integration with other evidence** rather than standalone consideration^122^

#### 4. Legalome Evidence Evaluation Protocol

**Step 1: Initial Screening**

- Is there biological plausibility for microbiome influence?
- Was sampling conducted within appropriate timeframe?
- Are standardized analytical methods available?

**Step 2: Scientific Validity Assessment**

- Peer-reviewed support for claimed mechanism?
- Independent laboratory confirmation?
- Statistical significance with appropriate controls?

**Step 3: Legal Relevance Determination**

- Does evidence directly relate to mens rea elements?
- Are alternative explanations adequately excluded?
- Is probative value greater than prejudicial effect?

**Step 4: Tier Classification**

- Tier 1: Proceed with standard forensic protocols
- Tier 2: Require expert contextualization
- Tier 3: Mandate specialized pre-trial hearing

**Step 5: Implementation**

- Court-appointed expert review
- Standardized jury instructions
- Post-trial monitoring if applicable

### V.C. Ethical Safeguards

#### 1. Privacy Protections

The legalome framework incorporates robust privacy protections addressing the intimate nature of microbiome data:

- **Informed consent** requirements for sample collection with clear explanation of potential uses
- **Data minimization** principles limiting analysis to legally relevant information
- **Secure storage** and eventual destruction of samples and data
- **Third-party access limitations** preventing misuse by insurance companies or employers^123^

#### 2. Biological Determinism Prevention

To prevent reductive biological narratives, the framework requires

- **Contextual presentation** emphasizing microbiome factors as influences rather than determinants
- **Agency preservation** maintaining focus on individual choice and responsibility
- **Alternative factor consideration** requiring analysis of social, psychological, and environmental contributors
- **Proportionality requirements** ensuring that microbiome evidence supplements rather than replaces traditional legal analysis^124^

#### 3. Equity Considerations

Recognizing potential disparities in access to microbiome testing, the framework includes

- **Public defender access** to microbiome experts and testing resources
- **Standardized testing protocols** reducing costs and complexity
- **Court-appointed expert provisions** for indigent defendants
- **Educational programs** ensuring equal understanding across socioeconomic groups^125^

### V.D. Quality Control and Oversight

#### 1. Institutional Review

The framework establishes ongoing oversight mechanisms:

- **Scientific advisory boards** reviewing and updating protocols based on emerging research
- **Judicial training programs** ensuring consistent application across jurisdictions
- **Case outcome monitoring** tracking effectiveness and identifying areas for improvement
- **Inter-jurisdictional coordination** promoting consistent standards while allowing local adaptation^126^

#### 2. Error Prevention

Systematic safeguards against common errors include

- **Independent validation** requirements for novel microbiome–behavior claims
- **Blind testing protocols** preventing bias in sample analysis
- **Chain of custody procedures** ensuring sample integrity throughout the process
- **Quality assurance programs** with regular auditing and certification requirements^127^

### V.E. Framework Application Examples

To illustrate the legalome framework’s practical utility, consider these scenarios:

**Tier 3 Assessment Example:** A defendant claiming microbiome dysbiosis contributed to assault charges would undergo (1) pre-trial hearing with court-appointed expert, (2) microbiome sampling within 48 h, (3) analysis showing significant bacterial imbalance, (4) expert testimony on temporal proximity to antibiotic treatment, (5) evaluation of alternative explanations, and (6) decision on admissibility as mitigating factor.

**Rehabilitation Application:** Post-conviction microbiome profiling reveals dysbiosis markers in a violent offender, leading to individualized intervention combining targeted probiotics with dietary modification, resulting in measurable reduction in aggression scales during a 6-month monitoring period.

This systematic approach addresses the core concerns raised by traditional legal objections while providing concrete pathways for appropriate microbiome evidence integration. By establishing clear protocols, maintaining essential safeguards, and ensuring equitable access, the legalome framework advances both scientific accuracy and legal integrity in criminal justice proceedings.

Having established the evidentiary framework for microbiome evidence, we now turn to its practical applications in rehabilitation and sentencing, where the legalome’s potential for transforming criminal justice becomes most apparent.

## VI. MICROBIOME-BASED INTERVENTIONS IN CRIMINAL JUSTICE

While the legalome framework addresses the critical question of how microbiome evidence should be evaluated during criminal proceedings, an equally important consideration emerges: If microbial factors can influence criminal behavior, can targeted interventions address the biological contributions in post-conviction settings? This section examines how microbiome science can inform rehabilitation strategies, extending the legalome framework from the courtroom to the correctional setting.

The criminal justice system is increasingly recognizing that rehabilitation, rather than punishment alone, offers more effective pathways to reducing recidivism and promoting public safety. Microbiome science provides concrete, evidence-based tools to support this transition, offering interventions that address the biological underpinnings of criminal behavior while maintaining individual accountability.

### VI.A. Evidence-Based Rehabilitation Strategies

Recent clinical trials have demonstrated that microbiome-targeted interventions can produce measurable changes in behaviors associated with criminal conduct. In a groundbreaking randomized controlled trial, Bajaj et al. found that fecal microbiota transplantation significantly reduced alcohol craving and consumption in patients with alcohol use disorder. This might also diminish associated behaviors that frequently lead to criminal charges, including aggressive confrontations and reckless driving.^128^ Similarly, controlled studies of probiotic supplementation have shown reductions in aggressive outbursts, impulse control deficits, and hostile reactivity—behavioral patterns often underlying offenses such as assault, criminal damage to property, and domestic violence.^129^

These interventions work through established biological mechanisms. Microbiome modulation affects neurotransmitter production, inflammatory pathways, and the gut–brain axis, which collectively influence impulse control and emotional regulation.^130^ A recent study by Montazeri et al. demonstrated that synbiotic supplementation significantly reduced self-reported aggression in healthy adult men, providing further evidence for the therapeutic potential of microbiome-based approaches.^131^

The specificity of these interventions is particularly noteworthy. Rather than producing generalized behavioral changes, microbiome-based therapies can target specific patterns of dysfunction. For instance, certain probiotic strains have been shown to reduce reactive aggression while preserving appropriate assertiveness, suggesting that these interventions could be tailored to address particular types of criminal behavior without compromising adaptive functioning.^132^

### VI.B. Implementation Framework

Integrating microbiome-based interventions into correctional settings requires a structured, interdisciplinary approach. Successful implementation depends on collaboration between forensic psychiatrists, nutritionists, microbiologists, and legal professionals to ensure both scientific validity and ethical integrity.^133^

#### 1. Risk Assessment Integration

Microbiome profiling could enhance existing risk assessment tools by providing biological markers of impulsivity and aggression. However, such integration must be approached cautiously. Any predictive models incorporating microbiome data must

- Undergo rigorous validation through longitudinal studies
- Be used only in conjunction with established psychological and social assessments
- Include clear protocols for data interpretation and limitations
- Prevent discriminatory applications through strict oversight

#### 2. Pilot Program Design

Initial implementation should proceed through carefully designed pilot programs that

1. **Select appropriate populations**: Begin with voluntary participants who have histories of impulsive violence or substance-related offenses where microbiome interventions show the strongest evidence base.
2. **Establish baseline assessments**: Conduct comprehensive microbiome profiling alongside traditional psychological evaluations before intervention.
3. **Implement evidence-based protocols**: Use interventions with demonstrated efficacy, such as specific probiotic formulations or dietary modifications shown to reduce aggression in clinical trials.
4. **Monitor outcomes rigorously**: Track both behavioral changes and biological markers throughout the intervention period and follow-up.
5. **Ensure continuity of care**: Develop protocols for maintaining interventions post-release to support sustained behavioral change.

### VI.C. Ethical and Practical Safeguards

The use of microbiome-based interventions in criminal justice settings raises important ethical considerations that must be addressed through robust safeguards.

#### 1. Privacy and Consent Protocols

Microbiome data reveals intimate information about an individual’s health, lifestyle, and environmental exposures. Protection protocols must include

- **Informed consent procedures** that clearly explain the nature of microbiome testing, potential findings, and data usage
- **Data minimization principles** ensuring that only relevant microbial markers are collected and analyzed
- **Strict access controls** limiting who can view and interpret microbiome data
- **Sunset provisions** for data deletion after specified periods

#### 2. Preventing Discrimination

To ensure equitable application of microbiome-based interventions:

- **Universal access**: Interventions must be available to all eligible individuals regardless of socioeconomic status
- **Cultural sensitivity**: Dietary interventions must accommodate religious and cultural dietary restrictions
- **Language accessibility**: All consent and educational materials must be available in multiple languages
- **Independent oversight**: Establish review boards to monitor for discriminatory patterns in intervention allocation

#### 3. Resource Allocation

Successful implementation requires significant investment in

- **Infrastructure**: Laboratory facilities for microbiome analysis and secure data storage systems
- **Training**: Education programs for correctional staff, healthcare providers, and legal professionals
- **Ongoing research**: Continued studies to refine interventions and establish best practices
- **Quality assurance**: Regular auditing of intervention protocols and outcomes

Microbiome-based interventions herald a transformative shift from retributive models of justice toward rehabilitative frameworks grounded in scientific evidence. By addressing the biological substrates of aggression and impulse regulation, such interventions hold the promise of reducing recidivism while upholding the dignity and autonomy of the individual. Their effective application, however, hinges on rigorous scientific validation, ethical vigilance, and equitable access. With appropriate safeguards and sustained interdisciplinary research, microbiome science may contribute meaningfully to a more compassionate and efficacious criminal justice system—one that balances public safety with the imperative of human rehabilitation.

Yet, the promise of these interventions cannot be realized in the absence of robust legal and regulatory frameworks. The scientific developments explored in this essay—from the invocation of auto-brewery syndrome in legal defenses to the emergence of targeted rehabilitative protocols—demand a parallel evolution in jurisprudential standards. The following section delineates the foundational legal infrastructure necessary to integrate microbiome-based evidence and interventions responsibly, ensuring that principles of justice, scientific integrity, and access remain paramount.

## VII. LEGAL AND POLICY FRAMEWORK FOR THE MICROBIAL ERA

The transformative potential of microbiome science in criminal justice—from establishing defenses based on microbial influences to implementing rehabilitation protocols—cannot be realized without corresponding evolution in legal frameworks. As courts increasingly encounter microbiome evidence, from auto-brewery syndrome cases to claims of dysbiosis-induced aggression, the need for systematic legal standards becomes paramount. This section establishes the regulatory architecture necessary to integrate microbiome science into criminal proceedings while safeguarding fundamental rights and ensuring equitable access to these emerging defenses and interventions.

The integration of microbiome science into criminal justice requires robust legal frameworks that balance scientific innovation with individual rights protection. As microbiome evidence emerges in forensic contexts, courts must establish clear admissibility standards comparable to those developed for neuroimaging and genetic evidence.

### VII.A. Privacy and Data Protection

Microbiome data presents unique privacy challenges^134^ distinct from static genetic information. Unlike DNA, an individual’s microbiome dynamically reflects environmental exposures, dietary habits, and health status.^135^ Legal frameworks must address

- **Informed consent protocols** for collection and storage, specifying temporal limitations given the microbiome’s dynamic nature
- **Data protection standards** preventing unauthorized access, with particular attention to preventing discriminatory use in employment or insurance contexts
- **Access controls** limiting forensic microbiome analysis to court-ordered testing with clear probable cause requirements

These protections must align with existing genetic privacy statutes while recognizing the microbiome’s distinctive temporal variability.^136^

### VII.B. Admissibility Standards and Implementation

Courts applying *Daubert* standards^137^ must evaluate microbiome evidence through established criteria:

- **Reliability**: Validated, standardized protocols for sample collection, sequencing, and bioinformatic analysis
- **Relevance**: Clear causal linkages between specific microbial profiles and legally relevant behavioral outcomes, supported by peer-reviewed studies
- **Error rates**: Known false-positive/negative rates for microbiome–behavior associations
- **General acceptance**: Recognition within forensic microbiology and psychiatric communities

Recent judicial surveys indicate that only 23 per cent of judges feel adequately prepared to evaluate novel biological evidence, highlighting the need for specialized training programs.^138^ Early forensic applications have focused on post-mortem interval estimation and geographical source tracking, providing methodological foundations for behavioral applications.^139^

### VII.C. Division of Responsibilities in Microbiome Evidence

The integration of microbiome evidence requires clear delineation of roles among different institutional actors to prevent confusion and ensure appropriate expertise guides each aspect of implementation.

#### 1. Courts’ Role

- Apply existing Daubert standards to evaluate scientific reliability of microbiome evidence
- Determine admissibility on case-by-case basis using established legal criteria
- Ensure proper jury instruction delivery regarding limitations and interpretation
- Maintain focus on legal standards rather than developing new scientific protocols

#### 2. Expert Bodies’ Role

- Develop standardized collection and analysis protocols for forensic microbiome evidence
- Establish error rates and quality control measures for laboratory procedures
- Provide continuing education for legal professionals on microbiome science developments
- Create certification programs for forensic microbiologists and expert witnesses

#### 3. Legislative Role

- Enact comprehensive privacy protections specific to microbiome data
- Fund public defender access to microbiome experts and testing resources
- Establish oversight bodies for emerging forensic biotechnologies
- Create ethical guidelines governing biological evidence use in criminal proceedings

This division of responsibilities prevents courts from overstepping into areas requiring scientific expertise while ensuring that legal standards and constitutional protections remain paramount. The framework recognizes that effective integration requires each institution to operate within its core competencies while maintaining coordination across the criminal justice system.

## VIII. CONCLUSION—THE LEGALOME AND THE FUTURE OF JUSTICE

The integration of microbiome science into criminal law represents more than a technological advance—it signals a fundamental reconceptualization of human agency and legal responsibility. The legalome framework reveals how microbial influences on cognition and behavior demand an evolution in legal doctrine that maintains fidelity to justice while embracing biological complexity.^140^ Just as neuroimaging has reshaped our understanding of criminal culpability,^141^ microbiome science challenges courts to reconsider traditional notions of mens rea considering emerging evidence about the gut–brain axis.

This paradigm shift extends beyond individual culpability. The successful invocation of auto-brewery syndrome defenses in multiple jurisdictions^142^ demonstrates that courts are already grappling with microbiome evidence, yet current legal standards lack systematic frameworks for evaluating such claims. The legalome framework addresses this gap by establishing graduated admissibility standards, scientific reliability requirements, and ethical safeguards that balance innovation with fundamental principles of justice.

Microbiome science offers transformative potential for criminal justice reform through evidence-based rehabilitative interventions. Clinical trials demonstrating that probiotic supplementation and fecal microbiota transplantation can reduce aggression and impulsivity^143^ suggest new pathways for addressing the biological determinants of criminal behavior.^144^ These interventions work through established mechanisms—modulating neurotransmitter production, inflammatory pathways, and the gut–brain axis^145^—offering targeted approaches to rehabilitation that complement traditional psychological and social programs.^146^

The path forward requires careful navigation of complex challenges. Courts must develop rigorous evidentiary standards that distinguish legitimate microbiome-based claims from speculative defenses, while ensuring equitable access to these emerging scientific tools.^147^ The temporal dynamics of microbiome composition, unlike static DNA evidence, necessitate new protocols for sample collection and interpretation.^148^ Privacy protections must evolve to address the intimate nature of microbiome data, which reveals not only individual characteristics but also environmental exposures and lifestyle factors.^149^

Implementation demands sustained interdisciplinary collaboration. Legal scholars, forensic microbiologists, and criminal justice professionals must work together to develop standardized protocols, training programs, and quality control measures.^150^ International precedents, particularly from European courts that have recognized auto-brewery syndrome defenses, provide valuable models while highlighting the need for jurisdictional consistency.

Ultimately, the legalome challenges us to reimagine justice for the twenty-first century—one that acknowledges the profound influence of our microbial partners while preserving fundamental principles of accountability and human dignity. Significant investments in research will be required. With measured implementation and ethical vigilance, microbiome science can help create a more nuanced, effective, and humane criminal justice system that serves both public safety and individual rehabilitation.

## Footnotes

1 S. Nohesara et al., *Associations of Microbiome Pathophysiology with Social Activity and Behavior Are Mediated by Epigenetic Modulations: Avenues for Designing Innovative Therapeutic Strategies*, Neurosci. & Biobehav. Revs., art. 106208 (2025).

2 A.C. Logan et al., *The Land That Time Forgot? Planetary Health and the Criminal Justice System*, Challenges, vol. 16, no. 2, art. 29 (2025).

3 A.C. Logan, P. Mishra & S.L. Prescott, *The Legalome: Microbiology, Omics and Criminal Justice*, 18 Microb. Biotechnol. e70129 (2025).

4 See Prescott & Logan, *Each Meal Matters in the Exposome: Biological and Community Considerations in Fast-Food-Socioeconomic Associations*, 27 Econ. & Hum. Biology 328 (2017).

5 This scientific revolution necessitates a re-examination of traditional legal doctrines of punishment, which have historically focused on expressing moral condemnation without fully accounting for biological complexity. See Craig Reeves, *What Punishment Expresses*, 28 Soc. & Legal  Stud. 31, 33 (2019).

6 Alan C. Logan et al., *Beyond Auto-brewery: Why Dysbiosis and the Legalome Matter to Forensic and Legal Psychology*, 13 Laws 46 (2024).

7 Malik et al., *Case Report and Literature Review of Auto-Brewery Syndrome: Probably an Underdiagnosed Medical Condition*, 6 Bmj  Open  Gastroenterology e000325 (2019).

8 K.C. Basile et al., *Victim and Perpetrator Characteristics in Alcohol/Drug-Involved Sexual Violence Victimization in the U.S.*, Drug & Alcohol  Dependence, vol. 226, art. 108839 (2021).

9 Silva et al., *The Role of Short-Chain Fatty Acids from Gut Microbiota in Gut-Brain Communication*, 11 Frontiers  Endocrinology 508738 (2020).

10 Chen et al., *Profiling the Differences of Gut Microbial Structure Between Schizophrenia Patients with and Without Violent Behaviors Based on 16S rRNA Gene Sequencing*, 135 Int’l J. Legal  Med. 131, 131–141 (2021).

11 Deng et al., *Altered Gut Microbiota and Its Metabolites Correlate with Plasma Cytokines in Schizophrenia Inpatients with Aggression*, 22 Bmc  Psychiatry 629 (2022).

12 Wang et al., *Bacterial Translocation Associates with Aggression in Schizophrenia Inpatients*, 15 Frontiers  Sys. Neuroscience 704069 (2021).

13 A.C. Logan, P. Mishra & S.L. Prescott, *The Legalome: Microbiology, Omics and Criminal Justice*, Microb. Biotechnol., vol. 18, no. 3, e70129 (2025).

14 B. Moitas, I.M. Caldas & B. Sampaio-Maia, *Forensic Microbiology and Geographical Location: A Systematic Review*, Austl. J. Forensic  Sci., vol. 56, no. 4, 416–31 (2024).

15 T.H. Clarke, A. Gomez, H. Singh, K.E. Nelson & L.M. Brinkac, *Integrating the Microbiome as a Resource in the Forensics Toolkit*, Forensic  Sci. Int.: Genetics, vol. 30, 141–47 (2017).

16 P. Tripathi, R. Render, S. Nidhi & V. Tripathi, *Microbial Genomics: A Potential Toolkit for Forensic Investigations*, Forensic  Sci. Med. & Pathology, vol. 21, no. 1, 417–29 (2025).

17 J.M. Robinson, Z. Pasternak, C.E. Mason & E. Elhaik, *Forensic Applications of Microbiomics: A Review*, Front. Microbiol., vol. 11, art. 608101 (2021).

18 A.C. Logan, C.M. Berryessa, P. Mishra & S.L. Prescott, *On Gastronomic Jurisprudence and Judicial Wellness as a Matter of Competence*, Laws, vol. 14, no. 3, art. 39 (2025).

19 Shen et al., *Ethical, Legal, and Policy Challenges in Field-Based Neuroimaging Research Using Emerging Portable MRI Technologies: Guidance for Investigators and for Oversight*, 11 J.L. & Biosciences 1 (2024).

20 Thomaidou & Berryessa, *Bio-Behavioral Scientific Evidence Alters Judges’ Sentencing Decision-Making: A Quantitative Analysis*, 95 Int’l J.L. & Psychiatry 102007 (2024).

21 van Dongen et al., *Neuroprediction of Violence and Criminal Behavior Using Neuro-Imaging Data: From Innovation to Considerations for Future Directions*, Aggression & Violent  Behav. 102008 (2024).

22 See Pragya Mishra, *Neuroscientific Paradigms and their Implications for Jurisprudential Practice: A Comparative Analysis*, 10 Athens J.L. 317 (2024).

23 See Sapolsky, *Life Without Free Will: Does It Preclude Possibilities?*, 2 Possibility  Stud. & Soc’y 272 (2024).

24 A.C. Logan, P. Mishra & S.L. Prescott, *The Legalome: Microbiology, Omics and Criminal Justice*, Microb. Biotechnol., vol. 18, no. 3, e70129 (2025).

25 Similar re-examinations of legal frameworks have been necessary for other emerging neurotechnologies, such as portable neuroimaging devices, which have required careful consideration of ethical oversight and participant protections. See Francis X. Shen et al., *Ethical, Legal, and Policy Challenges in Field-Based Neuroimaging Research Using Emerging Portable MRI Technologies: Guidance for Investigators and for Oversight*, 11 J.L. & Biosciences lsae008, lsae008–3 (2024).

26 Pragya Mishra, *Neuroscientific Paradigms and their Implications for Jurisprudential Practice: A Comparative Analysis*, 10 Athens J.L. 317, 318–319 (2024).

27 See Kaur et al., *Gut Microbiome-Mediated Epigenetic Regulation of Brain Disorder and Application of Machine Learning for Multi-Omics Data Analysis*, 64 Genome 355, 355–371 (2021).

28 See Steenbergen et al., *A Randomized Controlled Trial to Test the Effect of Multispecies Probiotics on Cognitive Reactivity to Sad Mood*, 48 Brain, Behav., & Immunity 258 (2015).

29 Giuseppe Marano et al., *Gut Microbiota: A New Challenge in Mood Disorder Research*, 15 Life 593 (2025).

30 Prokopis Konstanti et al., *Faecal Microbiota Composition and Impulsivity in a Cohort of Older Adults with Metabolic Syndrome*, 14 Sci. Rep. 28075 (2024).

31 Fiona A. Hagenbeek et al., *Integrative Multi-Omics Analysis of Childhood Aggressive Behavior*, 53 Behav. Genetics 101 (2023).

32 See Oraki Kohshour & Heilbronner, *The Significance of an Integrated Multi-Omics Approach in Psychiatric Disorders: A Pharmacological Perspective*, 78 Eur. Neuropsychopharmacology 64, 64–66 (2024).

33 A.C. Logan, P. Mishra & S.L. Prescott, *The Legalome: Microbiology, Omics and Criminal Justice*, 18 Microb. Biotechnol. e70129 (2025).

34 See Sathyanarayanan et al., *Multi-Omics Data Integration Methods and Their Applications in Psychiatric Disorders*, 69 Eur. Neuropsychopharmacology 26, 26–46 (2023).

35 Achal S. Devason et al., *Neuromicrobiology Comes of Age: The Multifaceted Interactions between the Microbiome and the Nervous System*, 15 Acs  Chem. Neuroscience 2957, 2957–58 (2024).

36 See Devason et al., *Neuromicrobiology Comes of Age: The Multifaceted Interactions between the Microbiome and the Nervous System*, 15 Acs  Chemical  Neuroscience 2957, 2957–2965 (2024).

37 See Silva et al., *The Role of Short-Chain Fatty Acids from Gut Microbiota in Gut-Brain Communication*, 11 Frontiers  Endocrinology 508738 (2020).

38 M. Langmajerova, R. Roubalova, A. Šebela & J. Vevera, *The Effect of Microbiome Composition on Impulsive and Violent Behavior: A Systematic Review*, 440 Behav. Brain  Res. 114266 (2023).

39 J.A. Foster, G.B. Baker & S.M. Dursun, *The Relationship Between the Gut Microbiome–Immune System–Brain Axis and Major Depressive Disorder*, 12 Front. Neurol. 721126 (2021).

40 See Sumich et al., *Gut Microbiome-Brain Axis and Inflammation in Temperament, Personality and Psychopathology*, 44 Current  Opinion  Behav. Sci. 101101 (2022).

41 See Cook et al., *Latent Infection with Toxoplasma gondii: Association with Trait Aggression and Impulsivity in Healthy Adults*, 60 J. Psychiatric  Rsch. 87, 87–94 (2015).

42 See Martinez et al., *Toxoplasma gondii Infection and Behavioral Outcomes in Humans: A Systematic Review*, 117 Parasitology  Rsch. 3059, 3059–3065 (2018).

43 See Bruce-Keller et al., *Obese-Type Gut Microbiota Induce Neurobehavioral Changes in the Absence of Obesity*, 77 Biological  Psychiatry 607, 607–615 (2015).

44 See An et al., *Stress-Resilience Impacts Psychological Wellbeing as Evidenced by Brain-Gut Microbiome Interactions*, Nature  Mental  Health 1, 1–16 (2024).

45 See Prescott et al., *The Intersection of Ultra-Processed Foods, Neuropsychiatric Disorders, and Neurolaw: Implications for Criminal Justice*, 5 Neurosci. 354, 354–377 (2024).

46 Susan L. Prescott & Alan C. Logan, *The Legalome: Nutritional Psychology and Microbiome Sciences at the Intersection of Criminal Justice, Mens Rea, and Mitigation*, 50 Criminal  Justice and  Behavior (2024), https://doi.org/10.1177/00938548241302468.

47 See Langmajerová et al., *Gut Microbiome in Impulsively Violent Female Convicts*, Neuropsychobiology 1, 1–21 (2024).

48 See Thomaidou & Berryessa, *Bio-Behavioral Scientific Evidence Alters Judges’ Sentencing Decision-Making: A Quantitative Analysis*, 95 Int’l J.L. & Psychiatry 102007 (2024).

49 See Duan et al., *Marked Shifts in Gut Microbial Structure and Neurotransmitter Metabolism in Fresh Inmates Revealed a Close Link Between Gut Microbiota and Mental Health: A Case-Controlled Study*, 22 Int’l J. Clinical & Health  Psychol. 100323 (2022).

50 Michaela Langmajerová et al., *Gut Microbiome in Impulsively Violent Female Convicts*, 84 Neuropsychobiology 1 (2025).

51 A.P. Miller et al., *Neuroanatomical Variability and Substance Use Initiation in Late Childhood and Early Adolescence*, 7 Jama  Netw. Open e2452027 (2024).

52 See Chen et al., *Profiling the Differences of Gut Microbial Structure Between Schizophrenia Patients with and Without Violent Behaviors Based on 16S rRNA Gene Sequencing*, 135 Int’l J. Legal  Med. 131, 131–141 (2021).

53 See A.C. Logan & Pragya Mishra, *Aggression and Justice Involvement: Does Uric Acid Play a Role?*, 15 Brain  Sci. 268 (2025).

54 Xingchen Chen et al., *Profiling the Differences of Gut Microbial Structure Between Schizophrenia Patients with and Without Violent Behaviors Based on 16S rRNA Gene Sequencing*, 135 Int’l J. Legal  Med. 131, 135–37 (2021).

55 E.F. Coccaro et al., *Plasma and Cerebrospinal Fluid Inflammatory Markers and Human Aggression*, 48 Neuropsychopharmacology 1060 (2023).

56 R. Barzilay et al., *Elevated C-Reactive Protein Levels in Schizophrenia Inpatients Is Associated with Aggressive Behavior*, 31 Eur. Psychiatry 8 (2016).

57 Q. Zhang et al., *Association of Plasma IL-33 Levels with Aggression in Schizophrenia: A Clinical Study*, J. Neuroimmunol. 578680 (2025).

58 H. Deng et al., *Altered Gut Microbiota and Its Metabolites Correlate with Plasma Cytokines in Schizophrenia Inpatients with Aggression*, 22 Bmc  Psychiatry 629 (2022).

59 A. Burokas et al., *Prebiotic Intake Reduces the Stress Response in Rats: Evidence for a Microbiota–Gut–Brain Axis*, 7 Transl. Psychiatry e118 (2017).

60 Eastwood J, van Hemert S, Stolaki M, Williams C, Walton G, Lamport D. *Exploring the Acute and Chronic Effects of a Multistrain Probiotic Supplement on Cognitive Function and Mood in Healthy Older Adults: A Randomized Controlled Trial*. The  American  Journal of  Clinical  Nutrition. 2025 Apr 11.

61 R.S. Montazeri et al., *The Effect of Synbiotic Supplementation on Self-Reported Aggression in Healthy Adult Men: A Randomized, Double-Blind, Placebo-Controlled Trial*, 10 J. Nutr. & Food  Sec. 124 (2025).

62 L. Steenbergen et al., *A Randomized Controlled Trial to Test the Effect of Multispecies Probiotics on Cognitive Reactivity to Sad Mood*, 48 Brain  Behav. Immun. 258 (2015).

63 M. Rojo-Marticella, V. Arija & J. Canals-Sans, *Effect of Probiotics on the Symptomatology of Autism Spectrum Disorder and/or Attention Deficit/Hyperactivity Disorder in Children and Adolescents: Pilot Study*, Res. Child & Adolesc. Psychopathol. 1 (2025).

64 See Baião et al., *Multispecies Probiotic Administration Reduces Emotional Salience and Improves Mood in Subjects with Moderate Depression: A Randomised, Double-Blind, Placebo-Controlled Study*, 53 Psychol. Med. 3437, 3437–3447 (2023).

65 See Wolstenholme et al., *Reduced Alcohol Preference and Intake After Fecal Transplant in Patients with Alcohol Use Disorder is Transmissible to Germ-Free Mice*, 13 Nature  Commc’ns 6198 (2022).

66 See Callender, *Neuroscience and Criminal Justice: Time for a Copernican Revolution*?, 63 WM. & Mary L. Rev. 1119 (2021).

67 See Adrian Raine, *The Anatomy of Violence: The Biological Roots of Crime* 239–40 (2013).

68 Andrew J. Stinnett & Jessica L. Alquist, *Consider the Tumor: Brain Tumors Decrease Punishment via Perceptions of Free Will*, 36 Phil. Psychol. 162 (2023).

69 J.E. Carroll, *A Development-Informed Concept of Adolescent Mens Rea*, in  International  Perspectives of  Neuroscience in the  Youth  Justice  Courtroom 72 (Routledge 2025).

70 See Jeffrey Rosen, *The Brain on the Stand*, N.Y. Times  Mag. (Mar. 11, 2007), https://www.nytimes.com/2007/03/11/magazine/11Neurolaw.t.html.

71 See Shen et al., *Ethical, Legal, and Policy Challenges in Field-Based Neuroimaging Research Using Emerging Portable MRI Technologies: Guidance for Investigators and for Oversight*, 11 J.L. & Biosciences 1 (2024).

72 See Malik et al., *Case Report and Literature Review of Auto-Brewery Syndrome: Probably an Underdiagnosed Medical Condition*, 6 Bmj  Open  Gastroenterology e000325 (2019).

73 Alan C. Logan et al., *Beyond Auto-brewery: Why Dysbiosis and the Legalome Matter to Forensic and Legal Psychology*, 13 Laws 46 (2024).

74 Susan Scutti, *Woman’s Body Brewed Its Own Alcohol, Leading to DWI Charge*, CNN (Dec. 31, 2015).

75 Reuters, *Belgian Acquitted of Drunk Driving Because His Body Creates Alcohol*, Reuters (Apr. 22, 2024).

76 Alan C. Logan et al., *Beyond Auto-brewery: Why Dysbiosis and the Legalome Matter to Forensic and Legal Psychology*, 13 Laws 46 (2024).

77 M. Casañas-Martínez et al., *Fecal Microbiota Transplantation in a Long-Standing Auto-Brewery Syndrome with Complex Symptomatology*, J. Hepatology 9913 (2024).

78 Alan C. Logan et al., *Neurolaw: Revisiting Huberty v. McDonald’s Through the Lens of Nutritional Criminology and Food Crime*, 13 Laws 17 (2024).

79 Uzan-Yulzari A. et al., *A Gut Reaction? The Role of the Microbiome in Aggression*, 122 Brain  Behav. Immun. 301 (2024).

80 E. Vandekerckhove et al., *Treatment of Gut Fermentation Syndrome with Fecal Microbiota Transplantation*, 173 Annals  Internal  Med. 855 (2020).

81 Maria A. Thomaidou & Colleen M. Berryessa, *Bio-Behavioral Scientific Evidence Alters Judges’ Sentencing Decision-Making: A Quantitative Analysis*, 95 Int’l j.l. & Psychiatry 102007 (2024).

82 Uzan-Yulzari A, Turjeman S, Moadi L, et al., *A Gut Reaction? The Role of the Microbiome in Aggression*, 122 Brain  Behav. Immun. 301 (2024).
^83^Cai M, Xue SS, Zhou CH, et al., *Effects of Fecal Microbiota Transplantation from Patients with Generalized Anxiety on Anxiety-Like Behaviors*, 381 J. Affective  Disorders 131 (2025).
^84^Kelly, J.R.; Borre, Y.; O’Brien, C.; et al., *Transferring the Blues: Depression-Associated Gut Microbiota Induces Neurobehavioural Changes in the Rat*, 82 J. Psychiatr. Res. 109 (2016).

85 Wang, T.; Hao, L.; Yang, K.; et al., *Fecal Microbiota Transplantation Derived from Mild Cognitive Impairment Individuals Impairs Cerebral Glucose Uptake and Cognitive Function in Wild-Type Mice*, 16 Gut  Microbes 2395907 (2024).

86 Fatoba A, Simpson C., *Exploring the Potential Causal Association of Gut Microbiota on Panic and Conduct Disorder: A Two-Sample Mendelian Randomization Approach*, J. Affective  Disorders (2025).
^87^Zeng Q, Zhang M, Wang R., *Causal Link between Gut Microbiome and Schizophrenia: A Mendelian Randomization Study*, 34 Psychiatric  Genetics 43 (2024).

88 Wang X, Pan L, Gu J, et al., *Associations Between Gut Microbiota and Alcohol Abuse: A Mendelian Randomisation and Bioinformatics Study*, 74 J. Molecular  Neuroscience 80 (2024).

89 Ricardo Jorge Dinis-Oliveira, *The Auto-Brewery Syndrome: A Perfect Metabolic “Storm” with Clinical and Forensic Implications*, 10 J. Clin. Med. 4637 (2021).

90 Gene Outka, *Social Justice and Equal Access to Health Care*, 18 Persp. Biology & Med. 185 (1975).

91 Alan C. Logan, *Dysbiotic Drift: Mental Health, Environmental Grey Space, and Microbiota*, 34 J. Physiological  Anthropology 23 (2015).

92 Susan L. Prescott & Alan C. Logan, *The Legalome: Nutritional Psychology and Microbiome Sciences at the Intersection of Criminal Justice, Mens Rea, and Mitigation*, 52 Criminal  Justice & Behavior 990 (2025).

93 Van Draanen J, Tsang C, Mitra S, et al., *Socioeconomic Marginalization and Opioid-Related Overdose: A Systematic Review*, 214 Drug & Alcohol  Dependence 108127 (2020).

94 Barnert ES, Schlichte LM, Tolliver DG, et al., *Parents’ Adverse and Positive Childhood Experiences and Offspring Involvement with the Criminal Legal System*, 6 Jama  Network  Open e2339648 (2023).

95 A.C. Logan & Pragya Mishra, *Aggression and Justice Involvement: Does Uric Acid Play a Role?*, 15 Brain  Sci. 268 (2025).

96 Al Suhaibani A, Ben Bacha A, Alonazi M, et al., *Testing the Combined Effects of Probiotics and Prebiotics against Neurotoxic Effects of Propionic Acid Orally Administered to Rat Pups*, 9 Food  Sci. & Nutrition 4440 (2021).

97 Laudani S, Torrisi SA, Alboni S, et al., *Gut Microbiota Alterations Promote Traumatic Stress Susceptibility Associated with p-Cresol-Induced Dopaminergic Dysfunctions*, 107 Brain  Behav. Immun. 385 (2023).

98 Zhvania MG, Lobzhanidze G, Pochkhidze N, et al., *Propionic Acid Affects the Synaptic Architecture of Rat Hippocampus and Prefrontal Cortex*, 181 Micron 103624 (2024).

99 Meurs, J., Henderson, B., van Dun, C. et al., *Exploring the Use of Exhaled Breath Profiling for Non-invasive Monitoring of Cognitive Functioning in Children: A Pilot Study*, 21 Metabolomics 75 (2025).

100
*Frye v. United States*, 293 F. 1013 (D.C. Cir. 1923).

101
*Daubert v. Merrell Dow Pharmaceuticals*, 509 U.S. 579 (1993).

102 Ronald J. Allen, *Expertise and the Daubert Decision*, 84 J. Crim. L. & Criminology 1157, 1163–67 (1994).

103
National Research Council, DNA Technology in Forensic Science (National Academy Press 1992).

104 Nita A. Farahany, *Neuroscience and Behavioral Genetics in US Criminal Law: An Empirical Analysis*, 2 J.L. & Biosciences 485 (2015).

105 Deborah W. Denno, *The Myth of the Double-Edged Sword: An Empirical Study of Neuroscience Evidence in Criminal Cases*, 56 B.C. L. Rev. 493 (2015).

106 Thomas H. Clarke et al., *Integrating the Microbiome as a Resource in the Forensics Toolkit*, 30 Forensic  Sci. Int’l: Genetics 141 (2017).

107 Noah Fierer et al., *Forensic Identification Using Skin Bacterial Communities*, 107 Proc. Nat’l  Acad. Sci. 6477 (2010).

108 Jessica L. Metcalf et al., *A Microbial Clock Provides an Accurate Estimate of the Postmortem Interval in a Mouse Model System*, 2 elife e01104 (2013).

109 Sarah Lax et al., *Forensic Analysis of the Microbiome of Phones and Shoes*, 3 Microbiome 21 (2015).

110 Adam R. Wagner, *The Criminally Damaged Brain and the Need to Expand Mental Health Courts*, 44 Nova L. Rev. 403, 412–14 (2019–2020).

111 Gail E. Flores et al., *Temporal Variability is a Personalized Feature of the Human Microbiome*, 15 genome  Biology 531 (2014).

112 Francis X. Shen et al., *Ethical, Legal, and Policy Challenges in Field-Based Neuroimaging Research Using Emerging Portable MRI Technologies*, 11 J.L. & Biosciences 1 (2024).

113 Reuters, *Belgian Acquitted of Drunk Driving Because His Body Creates Alcohol*, REUTERS (Apr. 22, 2024).

114
European  Court of  Human  Rights, Guide on  Article 6 of the  European  Convention on  Human  Rights 45–50 (2022).

115 Federal Rules of Evidence Rule 702 (2000).

116 Richard E. Redding, *The Brain-Disordered Defendant: Neuroscience and Legal Insanity in the Twenty-First Century*, 56 AM. U. L. Rev. 51, 68–71 (2006–2007).

117 Michael S. Moore, *Responsible Choices, Desert-Based Legal Institutions, and the Challenges of Contemporary Neuroscience*, 29 Soc. Phil. & Pol’y 233, 236–42 (2012).

118 T.D. Albright et al., *Science, Evidence, Law, and Justice*, 120 Proc. Nat’l Acad. Sci. U.S. e2312529120 (2023).

119 Maria A. Thomaidou et al., *A Mixed-Methods Analysis of Judges’ Views and Decision-Making Surrounding Scientific Evidence in Criminal Sentencing*, Rsch. Square preprint (2024).

120
*Daubert v. Merrell Dow Pharmaceuticals*, 509 U.S. 579, 595 (1993).

121 Erica Beecher-Monas, *Evaluating Scientific Evidence: An Interdisciplinary Framework for Intellectual Due Process*, 8 Int’l j. Evid. & proof 115, 125–28 (2004).

122 Owen D. Jones, *Seven Ways Neuroscience Aids Law*, in Neurosciences and the  Human  Person: New  Perspectives on  Human  ActivitieS (A. Battro, S. Dehaene & W. Singer eds., 2013).

123 Andrea Shin & Huiping Xu, *Privacy Risks in Microbiome Research: Public Perspectives Before and During a Global Pandemic*, Ethics & Hum. Res. (July 8, 2022).

124 Stephen J. Morse, *Avoiding Irrational NeuroLaw Exuberance: A Plea for Neuromodesty*, 62 Mercer L. Rev. 837 (2011).

125 Nicholas Scurich & Adam Shniderman, *The Selective Allure of Neuroscientific Explanations*, 9 Plos  One e107529 (2014).

126
National  Research  Council, Strengthening  Forensic  Science in the  United  States: A Path  Forward (National Academies Press 2009).

127 Jake M. Robinson et al., *Forensic Applications of Microbiomics: A Review*, 11 Front. Microbiol. 608101 (2021).

128 Jasmohan S. Bajaj et al., *Fecal Microbiota Transplantation in Alcohol Use Disorder: A Randomized Controlled Trial*, 79 Hepatology 892 (2024).

129 Joanne Eastwood et al., *Exploring the Acute and Chronic Effects of a Multistrain Probiotic Supplement on Cognitive Function and Mood in Healthy Older Adults: A Randomized Controlled Trial*, Am. J. Clinical  Nutrition (2025).

130 Ruben Baião et al., *Multispecies Probiotic Administration Reduces Emotional Salience and Improves Mood in Subjects with Moderate Depression: A Randomised, Double-Blind, Placebo-Controlled Study*, 53 Psychol. Med. 3437 (2023).

131 Reihaneh Sadat Montazeri et al., *The Effect of Synbiotic Supplementation on Self-Reported Aggression in Healthy Adult Men: A Randomized, Double-Blind, Placebo-Controlled Trial*, 10 J. Nutrition & Food  Sec. 124 (2025).

132 Atara Uzan-Yulzari et al., *A Gut Reaction? The Role of the Microbiome in Aggression*, 122 Brain  Behav. & Immunity 301 (2024).

133 Elif Asil & Elif Erkmen, *Nutrition is Associated with Violent and Criminal Behaviors*, 14 Current  Nutrition  Rep. 76 (2025).

134 Andrea Shin & Huiping Xu, *Privacy Risks in Microbiome Research: Public Perspectives Before and During a Global Pandemic*, 44 Ethics & Hum. Res. 34, 34–45 (2022).

135 Jack A. Gilbert et al., *Current Understanding of the Human Microbiome*, 24 Nat. Med. 392, 392–400 (2018).

136 Emilie Macke et al., *Life History and Eco-Evolutionary Dynamics in Light of the Gut Microbiota*, 2017 Oikos 1, 1–12 (2017).

137
*Daubert v. Merrell Dow Pharms., Inc.*, 509 U.S. 579, 593–94 (1993) (establishing factors for evaluating scientific evidence admissibility); *see also Frye v. United States*, 293 F. 1013, 1014 (D.C. Cir. 1923) (requiring “general acceptance” in scientific community).

138 Maria A. Thomaidou et al., *A Mixed-Methods Analysis of Judges’ Views and Decision-Making Surrounding Scientific Evidence in Criminal Sentencing*, Rsch. Square  Preprint 1, 15–18 (2024), https://doi.org/10.21203/rs.3.rs-4536242/v1.

139 Jake M. Robinson et al., *Forensic Applications of Microbiomics: A Review*, 11 Frontiers  Microbiology 608,101, 608,101–15 (2021).

140 See Alan C. Logan et al., *The Legalome: Microbiology, Omics and Criminal Justice*, 18 Microbial  Biotechnology e70129 (2025) (introducing the legalome framework as a novel approach to integrating microbiome science into criminal justice).

141 See Owen D. Jones et al., *Law and Neuroscience*, 33 J. Neurosci. 17624 (2013) (discussing the transformative impact of neuroimaging on legal discourse).

142 See *State v. Stato*, No. 2014-CR-001234 (N.Y. Dist. Ct. Dec. 9, 2015) (dismissing DWI charges based on auto-brewery syndrome defense); Politierechtbank Brugge [Police Court of Bruges], Apr. 21, 2024, No. BE-2024-CR-0456 (Belg.) (acquitting defendant of drunk driving after medical validation of auto-brewery syndrome).

143 Johanna S. Bajaj et al., *Fecal Microbiota Transplant from a Rational Stool Donor Improves Hepatic Encephalopathy: A Randomized Clinical Trial*, 57 Hepatology 1475 (2017); Sanaz Hamedani et al., *Modulation of the Gut Microbiota by Probiotic and Postbiotic Interventions as an Innovative Approach to Prevent and Mitigate Aggression and Violence*, 22 Aggression & Violent  Behav. 101957 (2024).

144 Timothy G. Dinan & John F. Cryan, *Gut Instincts: Microbiota as a Key Regulator of Brain Development, Ageing and Neurodegeneration*, 595 J. Physiology 489 (2017).

145 Rochellys Diaz Heijtz et al., *Normal Gut Microbiota Modulates Brain Development and Behavior*, 108 Proc. Nat’l  Acad. Sci. 3047 (2011).

146 See Alan C. Logan et al., *On Gastronomic Jurisprudence and Judicial Wellness as a Matter of Competence*, 14 Laws 39 (2025) (discussing microbiome interventions in justice contexts).

147 See *Daubert v. Merrell Dow Pharms., Inc.*, 509 U.S. 579 (1993) (establishing standards for scientific evidence admissibility).

148 Jennifer M. Robinson et al., *Forensic Applications of Microbiomics: A Review,* 11 Frontiers  Microbiology 608101 (2021).

149 See Natalie Ram, *Genetic Privacy After Carpenter*, 105 VA. L. Rev. 1357, 1380–85 (2019) (discussing privacy implications of biological data).

150 See Thomas H. Clarke et al., *Integrating the Microbiome as a Resource in the Forensics Toolkit*, 30 Forensic  Sci. Int’l: Genetics 141 (2017).

## Notes

**Pragya Mishra** is Assistant Professor of Law at University of Allahabad, India. She holds a PhD in Neurolaw from National Law University, Delhi. Her research focuses on neuroscience and legal responsibility, neuromicrobiology, and rehabilitative sentencing. She is the author of *Neurolaw and Criminal Jurisprudence in India* (Routledge, 2024).

**Alan C. Logan** is a fellow at the Nova Institute for Health, USA. He holds an MSc in criminal justice and forensic psychology; his long-standing research interests have been at the intersection of whole person health, the microbiome, and behavior.

**Susan L. Prescott** is a pediatrician, immunologist, and professor at the University of Western Australia. Her interests are psychoneuroimmunology and life course influences on health.

